# Family physicians' perspectives on practice guidelines related to cancer control

**DOI:** 10.1186/1471-2296-5-25

**Published:** 2004-11-15

**Authors:** Louise Zitzelsberger, Eva Grunfeld, Ian D Graham

**Affiliations:** 1 Clinical Epidemiology Program, Ottawa Health Research Institute, Ottawa Hospital, Canada; 2 Cancer Care Nova Scotia, Halifax, Canada; 3 School of Nursing, University of Ottawa, Canada

## Abstract

**Background:**

Family physicians (FPs) play an important role in cancer control. While FPs' attitudes towards, and use of guidelines in general have been explored, no study has looked at the needs of FPs with respect to guidelines for the continuum of cancer control. The objective of this study was to understand which guideline topics FPs consider important.

**Methods:**

Five group interviews were conducted by telephone with FPs from across Ontario, Canada. Transcripts were analyzed inductively. Content analysis identified emergent themes. Themes are illustrated by representative quotes taken from the transcripts.

**Results:**

The main areas where FPs felt guidelines were needed most included screening – a traditional area of responsibility for FPs – and treatment and follow-up – areas where they felt they lacked the knowledge to best support patients. Confusion over best practice when faced with conflicting guidelines varied according to disease site. FPs defined good guideline formats; the most often cited forms of presentation were tear-off sheets to use interactively with patients, or a binder. Computer-based dissemination was acknowledged as the best way of widely distributing material that needs frequent updates. However, until computer use is a common aspect of practice, mail was considered the most viable method of dissemination. Guidelines designed for use by patients were supported by FPs.

**Conclusions:**

Preferred guideline topics, format, dissemination methods and role of patient guidelines identified by FPs in this study reflect the nature of their practice situations. Guideline developers and those supporting use of evidence-based guidelines (e.g., Canadian Strategy for Cancer Control) have a responsibility to ensure that FPs are provided with the resources they identify as important, and to provide them in a format that will best support their use.

## Background

Family physicians (FPs) play an important role in cancer control. Their traditional involvement has been primarily focused on opposite ends of the cancer control continuum: prevention, screening and diagnosis at the beginning of the continuum, and provision of palliative care at the other end. Treatment and follow-up have typically been the responsibility of secondary or tertiary care physicians.

There are indications that FPs would like their traditional roles to include involvement in treatment and follow-up [1,2], and that they are, in fact, becoming more involved in these areas [3,4]. A survey of Canadian FPs showed that they felt a significant proportion of long term follow-up care could be transferred to the FP decreasing the burden on consultants [5]. For example, in a randomized trial of routine follow-up of breast cancer patients, the care provided by FPs was found to be equivalent to that of an oncology specialist [6]. A concern, however, with increased involvement is that FPs receive little oncological training in medical school [4], and thus, are not adequately prepared for involvement in certain aspects of cancer care particularly as treatment practices change as new evidence emerges.

Clinical practice guidelines (CPGs) can provide FPs with information and guidance on evidence-based best practices. While FP attitudes on the use of CPGs have been shown on the whole to be positive [7-11], FPs have suggested that involvement of FPs in the development of guidelines that take into account the nature of primary care would improve their uptake among FPs [11-14]. Dowswell et al[11] suggested that rather than ask how to get physicians to follow guidelines, it would be more productive to ask physicians about their information needs and how they would like them met.

A broad partnership of key stakeholders known as the Canadian Strategy for Cancer Control (CSCC) is currently working towards a national cancer control strategy. The CSCC initiative builds upon previous work done in Canada, and has the goal of developing, adopting and implementing a national strategy. A focus on "Guidelines and Standards" is one of the CSCC's top priorities with an aim to establish mechanisms and improve capacity for collaborative guideline and standards development [15]. In order to ensure wise use of resources, it will be important to ascertain the needs of FPs in Canada with respect to CPGs relevant to cancer control. While many studies have focused on cancer screening [16-18] and palliative care [19,20], to our knowledge, no study has looked at what FPs consider important in terms of content, format and dissemination of guidelines related to cancer control activities. The objective of this study was to learn the views of Ontario FPs for the furtherance of cancer control efforts by the provincial cancer agency, Cancer Care Ontario. The information derived from the study is also of benefit for national cancer control efforts through the CSCC.

## Methods

In order to learn FPs' views on guidelines on cancer control, a qualitative methodology was considered the most appropriate. To facilitate FPs' involvement (e.g., eliminate travel and time barriers) and ensure representation of FPs practicing in remote areas, the most feasible option was to hold interviews by teleconference [21]. Thus, we conducted the process as key informant interviews involving between two and four participants [22,23] and using a semi-structured interview format.

With respect to recruitment, FPs from various regions in Ontario identified through the Canadian Medical Directory were recruited via an information letter and follow-up telephone call. In addition, FP colleagues identified potential participants or individuals who might suggest potential participants. Sampling was purposeful [23]. We felt that it was important to involve FPs from the different regions in Ontario (northern, eastern, central east, southwest and central west), as well as have both urban and rural representation, as needs with respect to guidelines may differ based on these characteristics. Once a commitment to participate was made, a time and date for the teleconference were established. FPs were then faxed a consent form and a list of questions that would be asked during the interview.

To allow for in-depth discussion, three cancer disease sites were selected: lung, colorectal and cervix. The first two were included because of high incidence; the latter because conflicting screening guidelines [21] currently exist [24-27]. Questions focused on preferred topics for guidelines along the cancer control continuum, preferred format and method of dissemination of guidelines, and perspectives on guidelines written for patients. Questions were tested for clarity and coverage of important issues in a pilot session involving four FPs and the moderator (LZ). As a result of the pilot, it was decided that four participants were the maximum number for each session to ensure that the session was not too long – an important consideration for physicians with busy schedules.

Five group interviews were held. There was a minimum of 2 and maximum of 4 FP participants in each session. Each of the five interview sessions lasted between 45 and 60 minutes. In order to accommodate FPs' schedules, three of the five interviews were held in the early evening, and two in the morning. FPs were asked to select either a lottery ticket or telephone card as a small acknowledgement of their participation. Each session was led by the same individual (LZ), an experienced qualitative researcher. Interviews were audio-taped.

Audio tapes were transcribed immediately after each interview, and underwent a preliminary analysis allowing for emergent issues or ideas to be explored in future sessions. Transcripts were read by one of the researchers (LZ); latent content analysis (coding and classification into themes) was done manually [22]. As each question addressed a specific topic, the question topics themselves acted as broad organizing categories. Relevant transcript sections were marked and assigned code words. Codes of similar type and content were combined into sub-categories within each question topic [28].

A second researcher (EG) was given two transcripts to read and code independently using the list of codes and categories. Coding was compared. Where necessary, definitions of code words were refined and categories expanded upon [29].

Disagreements and differences were resolved through discussion and data was re-examined where necessary [30]. Representative quotes were selected from the transcripts in order to illustrate key issues raised by the participants.

Ethical approval for this study was obtained from the Ottawa Hospital Research Ethics Board.

## Results

Of the 13 physicians participating in the study, seven were male and six were female; five practiced in a rural setting, the remainder in an urban setting. The majority (9/13) were in group practice. Approximately 5% of FPs contacted agreed to participate in the study. Overall, topics raised by urban and rural FPs were similar except with respect to guidelines on cancer treatment.

### Guideline topics

Using lung, colorectal and cervical cancers as exemplars, FPs were asked for which topics along the cancer control continuum (i.e., prevention, screening, diagnosis, treatment, follow-up, or palliation) they most wanted guidelines. Screening was the topic most frequently mentioned, although reasons behind requests for screening guidelines differed for each disease site. For lung cancer, there are currently no evidence-based screening tools or maneuvers, and no screening guidelines. FPs were uncertain whether to routinely screen for lung cancer in their practices. For colorectal cancer, on the other hand, there are a number of screening tools and a number of recommendations made by different organizations. Conflicting guidelines resulted in FPs being uncertain about what to do in practice.

I'm certainly much less certain of the area of screening for colorectal cancer. I mean there's a lot of different guidelines out there, it depends on who you read and I regard that as an area very much in flux...I still remain a bit confused as to who should have what. (FP_6_)

FPs also commented that a similar confusion was prevalent with regard to screening guidelines for cervical cancer.

After three normal [screens], every two years and discontinue at the age of 70, that's what it says. And then this other one says start at age 18, and after three normals then do it every three years except high risk patients should have annual smears...the American College of OB/GYN recommends its smears always continue annually. The American Cancer Society and the Canadian Task Force recommends screening until age 65 and 69 respectively. So, it's a dog's breakfast. (FP_5_)

However, in comparison to colorectal cancer screening, there was much less ambiguity about what to do in practice. FPs readily adapted cervical cancer screening guidelines to suit individual patient situations or demands.

I would say I have people who I am willing to see every 3 years because I feel quite confident that they'll be back; they're good at keeping up and the ones that I'm more uncertain about in terms of their follow up, I'll make sure I do it more frequently just in case...For me it varies very much between 1 and 3 years and it is very much a decision of my own. (FP_11_)

In discussing the issue of conflicting guidelines, FPs raised the point that where more than one guideline exists, the credibility of all come into question. For example, if there are multiple guidelines on a topic, can any be the 'right' one to use?

FPs were also very interested in treatment guidelines. Interest centered around two situations: decision making with patients, and dealing with side-effects of treatment. In the first, FPs wanted to know about treatment available to their patients diagnosed with cancer. They saw their role as helping patients and families make informed choices. As such, they wanted information on treatment goals, survival rates for different treatments, quality of life issues as well as risk of and dealing with potential side-effects.

A lot of the patients I have who go out to a cancer clinic come back and make sure I agree with what they're choosing and the problem is I don't have the information to be able to even aid them in making their decision....So if we had a little bit more information than those flow charts that are 'yes/no' to say [this] is the most recent information for survival rates...that kind of thing would be helpful. (FP_7_)

The second area regarding treatment was raised by rural physicians who often saw cancer patients in emergency departments when, for example, patients were home between chemotherapy cycles. FPs mentioned the difficulty caring for patients when they knew little about their treatment plan.

Areas where we really need specific evidence-based guidelines are in treatment and follow-up. I mean, although the patient may disappear to the cancer clinic...they certainly do show up in emergency, and sometimes the husband or wife calls us as well and says, "Well you know they're getting this drug or they're getting this radiation, they're really sick and what do you think about this?" And if we don't even know what they're getting or what the potential side-effects are, it's really hard to be helpful. So, we need specific guidelines. (FP_5_)

Rural physicians were also interested in guidelines for follow-up.

...we are going to be doing more and more of our own follow-up, that's the trend, that's the next century, so we need good guidelines. (FP_5_)

Guidelines were seen by some FPs as a potential communication device between cancer centres and community-based FPs. They suggested that guidelines could be sent to the FP from the cancer centre and include notations by oncologists regarding individual patients. In this way, FPs would feel they had the tools to provide on-going support and care for patients in the treatment or follow-up stages.

### Format and dissemination

Two themes were identified related to guideline format: format aspects and presentation. Regarding format, Table 1 presents FPs' 'definition' of what attributes comprise a good guideline. The outstanding features requested were a combination of brevity, and formatted in such a way that FPs were able to quickly identify relevant content.

**Table 1:** Components of a 'Good' Guideline

Dated
Has a clearly defined, reputable source
Involves FPs in the creation process to ensure its clinical practicality
Not too text based (graphics, tables, flowcharts)
Clear, non-ambiguous recommendations
Well organized
Clearly graded as to levels of evidence
One guideline from one authorative body (to reduce confusion)
Readable in a few minutes
Designed so that FPs will use the guideline frequently and become familiar with it
One to two pages long

The most popular forms of presentation suggested by FPs were a binder that would be easy to update, and tear-off sheets that could be given to the patient but which also provided a review opportunity for the physician through the act of explaining the guideline to the patient. CD ROMs, posters or software packages (guidelines and a recall system for screening) were other suggestions.

I like [the] idea of the tear off sheet to use in discussion with patients. I think guidelines are only useful to the extent that we can go over them again and again and actually be familiar with them ourselves, rather than just having them tucked in a big binder with many other guidelines. So, something that can be used with the patient. (FP_3_)

Computer-based dissemination was acknowledged as the best way of distributing material widely and addressing the difficulties and expense of updating material. Presentation by local leaders, CME, fax, small group meetings, and mail were other suggestions. While computers were mentioned most often, FPs emphasized that information needs to be widely disseminated to **all **physicians. For this reason, mail was still seen as the most viable form of dissemination.

### Patient guidelines

All FPs agreed that guidelines written for patients would be useful, although there was concern that they should be written very clearly, only be available for topics for which there is good evidence, and not be conflicting. They felt patient guidelines would be useful in that they would act as an added voice, giving weight to the FP's recommendation. Guidelines were also seen as useful in countering misinformation brought in by patients (e.g., from the Internet) to the consultation. On the whole, FPs felt that the more information patients had, the better. Three FPs felt that guidelines would encourage patients to take responsibility for their own care; patients could remind their FP if they were due for screening.

So I think the biggest effort is to establish the proper guidelines that are accepted by a group of authorities in Canada and then that would make it easier for me to say, "Well, look, this is the actual guideline that is the result of a great deal of research and in fact you really don't need that mammogram at the age of 40"...I think there has to be an effort to make sure that patients are not given conflicting guidelines. (FP_8_)

In terms of content, FPs felt the guideline should echo the FP message. In addition to the tear-off sheets mentioned earlier, ideas for presentation included an educational message played on the telephone when a patient calls, or video messages broadcast on office televisions.

## Discussion

FPs have traditionally been responsible for prevention, screening, and early detection, and palliative care. Of the topics along the cancer control continuum, screening guidelines were most frequently identified by FPs in this study. Screening for cancer is primarily the responsibility of FPs who need to stay informed of changes or conflicts in recommendations. British general practitioners, when interviewed about use of guidelines, said that they referred to guidelines for cases that they encountered either most commonly or most rarely in practice [12].

FPs' preferences for screening guidelines addressed two different information needs. In the case of lung cancer, where a familiar screening maneuver was not recommended (i.e., chest x-ray), FPs wanted guidelines that addressed what they should do for routine screening. In the case of colorectal cancer, FPs received conflicting messages about screening, and sought guidance as to which recommendation to use.

One barrier to guideline use is if guidelines are considered controversial [21,31,32]. FPs identified conflicts in recommendations for colorectal and cervical cancer screening. However, they expressed less difficulty in making decisions regarding cervical cancer screening for their patients in comparison with colorectal cancer screening. This may be because cervical cancer screening is a long established practice with good evidence of benefit. Differences between guidelines for cervical cancer relate to the length of interval between routine screening [24,25]. Conversely, colorectal screening is a new practice for which there have been long standing recommendations *against *routine screening. Currently, differences in recommendations relate to the type of screening maneuver [26,27].

Decisions regarding screening practice also depended on patient factors such as a patient's motivation to adhere to a screening routine. Physician factors (e.g., perceptions of guidelines and clinical experience) and patient factors have been identified as two of the three determinants of a decision making model for cancer screening in the case of unclear or controversial guidelines. The third determinant was the perceived quality of the physician-patient relationship and the clarity of the recommendation being discussed [21].

As with Feightner et al.[33], FPs in this study encouraged development of patient versions of FP guidelines. Guidelines were seen as an opportunity to counter misinformation brought in by patients and also as a means having of both physician and patient participate in the patient's care. The literature with respect to improving cancer screening practice shows that interventions that target both the physician and the patient have the greatest impact [34].

While the FPs in this study play a role in treatment and follow-up, they do not feel they have the information they need to help their patients. Practice location appears to influence the type of treatment information FPs want, although generalizations can not be made on the basis of the qualitative methods used in this study. In urban settings, FPs' roles along the continuum of cancer care principally involve prevention, screening, diagnosis, and palliation. In addition to the above roles, rural physicians are involved in treatment and follow-up, and this involvement is perceived as increasing in the future. Consequently, they state that guidelines on treatment and follow-up would be helpful. Rural physicians participating in a Canada-wide focus group and interview study on FP-oncologist communication also indicated a need for treatment and follow-up information [2].

While FPs expressed a need for guidelines on cancer follow-up, several guidelines have been published. In the case of colorectal cancer for example, a guideline has been published in Ontario under the auspices of Cancer Care Ontario [35]. The challenges with respect to guidelines that are not created specifically for FPs include a need to find the best ways of making FPs aware of the existence of such guidelines, and also to provide implementation strategies geared towards the FP practice [36-39].

FPs in this study preferred guidelines in a paper format disseminated by mail rather than electronically. The 2001 Janus survey conducted by the College of Family Physicians of Canada found that only approximately one quarter of FPs across Canada have access to and use computerized CPGs in their office [40]. The FPs in this study prefer a format that they could use interactively with patients. Recurrent use with patients was seen as a way of helping FPs assimilate the knowledge. Guidelines as a 'look-up' resource and as a general educational tool could become part of the general practitioner's knowledge base [12].

The findings of this study are limited by several factors. The purpose of the study was to gather information on FPs' perspectives on guidelines along the cancer control continuum. As with all qualitative research, the results of the study are not generalizable beyond the sample. However, the intent of sampling in qualitative research is to identify key informants who will illuminate particular aspects of the research topic [23]. FPs agreeing to participate in this study are likely those who have a strong interest in guidelines. The perspectives shared by FPs offer insight into the guideline topics, format, and dissemination of guidelines that FPs consider important in caring for their patients with cancer.

Further research should focus on identifying the guideline needs of a larger, nation-wide sample of Canadian FPs to ensure that efforts by initiatives such as the CSCC result in CPGs that will be both viewed positively and adopted by FPs. However, perception of the value of a guideline is not enough to ensure adoption. While much effort has gone into guideline development, the focus on dissemination has been through traditional dissemination strategies (e.g., publication in professional journals). Knowledge transfer is known to be more complex [41] requiring multifaceted strategies to encourage adoption and taking into account the environment, the potential user and characteristics of the innovation (e.g., guideline) [36,42,43,39,38].

## Conclusion

Guideline topics, format, dissemination, and patient guidelines discussed by FPs in this study reflect their particular practice situations. FPs' strongest preferences were for guidelines on cancer screening, followed by guidelines on treatment that would help them support and provide care for patients. The conflicting messages of some guidelines did not necessarily make decision making problematic for FPs. Rather, it was the reason behind the conflict that created difficulties. FPs saw patient guidelines as an educational tool for both themselves and their patient. Guidelines on treatment and follow-up are available, although they are generally geared towards specialists not FPs. This suggests a need for FP versions to be created. Challenges for Canadian guideline developers/implementers include not only ensuring that the evolving needs of FPs are met, but also that they address the needs of FPs with respect to how that information is formatted, delivered to FPs and how FPs are supported in its use.

## Competing interests

Dr. Graham is a CIHR New Investigator.

## Authors' contributions

LZ participated in the design of the study, conducted the interviews and analysis, and drafted the manuscript. EG participated in the design of the study, the analysis, and contributed to the manuscript. IG participated in the design of the study, and assisted with the pilot test of interview questions. All authors read and approved the final manuscript.

## Pre-publication history

The pre-publication history for this paper can be accessed here:


